# Laparoscopic Hernia Repair versus Open Herniotomy in Children: A Controlled Randomized Study

**DOI:** 10.1155/2012/484135

**Published:** 2012-12-27

**Authors:** Rafik Shalaby, Refaat Ibrahem, Mohamed Shahin, Abdelaziz Yehya, Mohamed Abdalrazek, Ibrahim Alsayaad, Maged Ali Shouker

**Affiliations:** ^1^Pediatric Surgery Unit, Al-Azhar University Hospitals, Cairo, Egypt; ^2^Pediatric Surgery Unit, Al-Azhar University Hospitals, Damietta, Egypt; ^3^Diagnostic Imaging Department, Al-Azhar University Hospitals, Cairo, Egypt

## Abstract

*Background*. Laparoscopic hernia repair in infancy and childhood is still debatable. The objective of this study is to compare laparoscopic assisted hernia repair versus open herniotomy as regards operative time, hospital stay, postoperative hydrocele formation, recurrence rate, iatrogenic ascent of the testis, testicular atrophy, and cosmetic results. *Patients and Methods*. Two hundred and fifty patients with inguinal hernia were randomized into two equal groups. Group A was subjected to laparoscopic inguinal hernia repair. Group B was subjected to open herniotomy. The demographic data were matched between both groups. Assessment of the testicular volume and duplex assessment in preoperative, early, and late postoperative periods were done. *Results*. All cases were completed successfully without conversion. The mean operative time for group A was 7.6 ± 3.5 minutes, 9.2 ± 4.6 minutes and 11.4 ± 2.7 minutes, for unilateral hernia, unilateral hernia in obese child, and bilateral hernia, respectively. The recurrence rate was 0.8% in group A, whereas in group B the recurrence rate was 2.4%. *Conclusion*. Laparoscopic hernia repair by RN is an effective line of hernia repair. It resulted in marked reduction of operative time, low rate of recurrence, no testicular atrophy, no iatrogenic ascent of the testis, and excellent cosmetic results.

## 1. Introduction

Inguinal hernia (IH) repair is one of the most frequently performed surgical procedures in infants and children. Open herniotomy is its standard treatment against which all alternative modalities of treatment are evaluated. It is credited with being easy to perform, having a high success rate, and low rate of complications. However, recently, many centers routinely perform laparoscopic hernia repair in children and there have been numerous reports describing various laparoscopic techniques rather than the traditional open approach [1–4].

Reported advantages of laparoscopic hernia repair include excellent visual exposure, minimal dissection, less complications, comparable recurrence rates, and improved cosmetic results compared with the traditional open approach. In addition, laparoscopic hernia repair also allows contralateral patent process vaginalis (PPV) hernias to be defined and repaired in the same operation [5–7].

Randomized control study of laparoscopic hernia repair versus OH in pediatrics is rare in the literature [8–10]. This paper presents a big series and describes a new technique which is the use of Reverdin Needle (RN) in laparoscopic hernia repair in comparison with OH, to the best of our knowledge, this technique has not been reported before. So, this prospective randomized controlled study was conducted to compare laparoscopic assisted hernia repair by RN with OH in infancy and childhood as regards operative time, hospital stay, postoperative hydrocele formation, recurrence rate, iatrogenic ascent of the testis, testicular atrophy, and cosmetic results.

## 2. Patients and Methods

**Figure 1:**
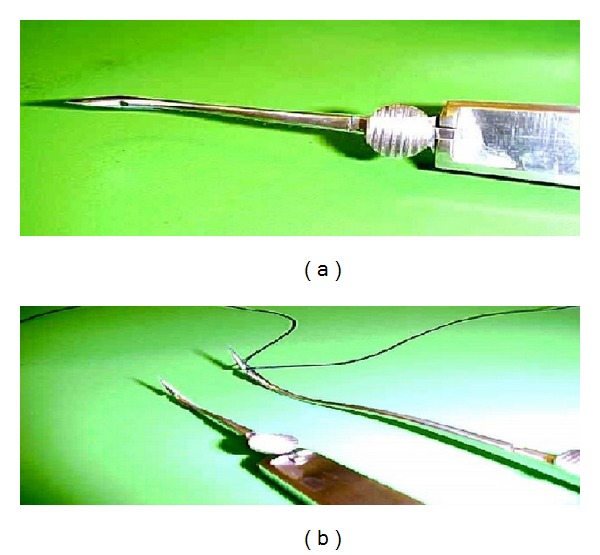
Reverdin needle.

A prospective randomized controlled study was carried out in the Pediatric Surgery Unit of Al-Azhar University Hospitals and 2 private hospitals, over four-year period. The study was approved by our ethical committee. Two hundred and fifty patients with IH were randomized into two equal groups by a random-number table sequence after a written informed parental consent was obtained. Group A (*n* = 125) was subjected to laparoscopic assisted inguinal hernia repair by RN (Figure 1) (Martin Medizin Technik, Tuttlingen, Germany). Group B (*n* = 125) was subjected to open herniotomy (OH). The demographic data were matched between both groups (Table 1). Inclusion criteria included bilateral inguinal hernia, recurrent hernia, hernia in obese child, inguinal hernia with umbilical hernia and hernia on ipsilateral with questionable contralateral side. Exclusion criteria included unilateral inguinal hernia in nonobese child and hernia with undescended testicles. The main outcome measurements were operative time, hospital stay, postoperative hydrocele formation, recurrence rate, iatrogenic ascent of the testis, testicular atrophy, and cosmetic results. All children were subjected to full history taking, thorough clinical examination, routine laboratory investigations, and inguinoscrotal U/S. Testicular size and perfusion of male cases (*n* = 179) were evaluated in the preoperative, early postoperative (within 48 hours of surgery), and late postoperative periods (6 months after surgery) using Gray-scale ultrasonography, and Doppler ultrasound (DUS) (both duplex and power Doppler mode). (Sonoline Antaris, Siemens, Medical Corporation U/S Erlangen, Germany). The patients were examined with a 7.5 MHz linear, phased-array transducer. Both testes were scanned in transverse and longitudinal planes while the patient was in the supine position, and sedation was used when required in the form of paracetamol suppository. The testis on the unaffected side (in unilateral cases) was scanned first to optimize the Doppler settings for assessment of slow blood flow in the testis.

The volume of testis on both sides was calculated using the ellipsoid formula (volume = 0.523 × *D*_1_ × *D*_2_ × *D*_3_), where *D*_1_, *D*_2_, and *D*_3_ were the maximally measured longitudinal, anteroposterior, and transverse diameters.

The ratio *v* was defined as *v* = testicular volume of the operated side (postoperatively)/testicular volume of the same side (preoperatively). Volume of the testis and the ratio *v* were calculated during the preoperative and late postoperative examinations.

Criteria of testicular atrophy were defined as 75% reduction in estimated testicular volume, ratio *v* less than 75%, and resistive index (RI) more than 0.7.

All operations were done by the first three authors, and a senior resident holds the camera. In group A, after induction of general endotracheal tube anesthesia, the patient was placed supine in Trendelenburg's position. Insertion of the main umbilical port was accomplished by the open method. Pneumoperitoneum was established to a pressure of 8 to 12 mm Hg.

**Figure 2:**
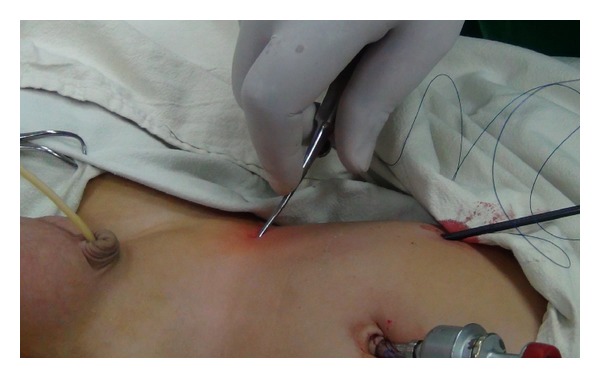
Insertion of RN on the right side.

Laparoscopy was used for initial visualization of the pelvis and IIRs on both sides. Laparoscopic hernia repair was done according to the technique described by Shalaby et al., 2006, with some modifications [11]. A 3 mm Maryland forceps, holding the tip of a 3/0 Prolene thread, was inserted into the abdomen without trocar at the lateral border of the rectus muscle just above the level of the umbilicus leaving the long end of the thread outside the abdomen (Figure 2).

A stab incision of the skin was done 2 cm above and lateral to the IIR on the right side, and 2 cm above and medial to the IIR on the left side and RN was inserted into the abdominal cavity (Figure 2). The needle was manipulated to pierce the peritoneum at 3 O'clock on IIR and was advanced to pass through the lower margin of IIR under the peritoneum and in front of the spermatic vessels and vas to pierce the peritoneum at 9 O'clock on the IIR. Care was taken to avoid injury of the spermatic vessels, and vas by grasping and lifting the peritoneum away from the vas and vessels and the RN was seen all the time beneath the peritoneum (needle sign). Then, the side of the hole of RN was opened and the thread hold by Maryland was inserted inside it. Then, the side of the hole of RN was closed, and the needle was withdrawn backward in the same path till reaching the starting point at 3 O'clock. Then, RN mounted by the thread was reinserted again at 3 O'clock and was advanced along the upper margin of the IIR beneath the peritoneum and fascia transversalis to come out from the same opening at 9 O'clock where the short end of the thread was withdrawn out of RN and pulled outside the abdominal cavity for extracorporeal suture tie. Before tightening the knot, the scrotum was squeezed and the intraperitoneal pressure was released to expel the gas in the hernial sac.

A contralateral internal ring with a patent processus vaginalis (more than 2 mm) was regarded as a possible cause of developing clinical hernia and repaired at the same time [7]. The skin incisions were closed with Steri-strips.

In group B, OH was done through an inguinal skin crease incision. High ligation of the sac was performed using 4/0, 3/0 absorbable (Monocryl) suture. The distal sac was slit open to prevent postoperative hydrocele formation. The wound was closed in layers, using absorbable suture.

All patients were followed up in the out-patient clinic after 7 days, 2 weeks, 6 months, 1 year, and 2 years. Parents were advised to contact the department of pediatric surgery, if there were any concerns in the immediate postoperative period.

## 3. Statistical Analysis

The collected data were organized, tabulated, and statistically analyzed using Statistical Package for Social Science (SPSS) version 16 (SPSS Inc., USA). Qualitative data, frequency, and percent distribution were calculated, and Chi square test was used for comparison between groups. Quantitative data, mean, standard deviation (SD), and range were calculated, and for comparison between two groups, the independent samples (*t*) test was used. For interpretation of results, *P* < 0.05 was considered significant.

## 4. Results

Two hundred and fifty patients with IH were operated upon by 2 different techniques. Group A (*n* = 125) was subjected to laparoscopic assisted inguinal hernia repair by RN. Group B (*n* = 125) was subjected to OH. They were 179 males and 71 females. The youngest was 5 months and the oldest was 96 months, given an overall mean age of 61.56 ± 28.32 months. All procedures of group A were completed laparoscopically without any conversion. No intraoperative complications occurred during this study.

In group A the patients resumed normal activities within 6 hours after surgery, whereas in patients of group B they resumed normal activities within 10 hours. All patients had uneventful postoperative recoveries and were discharged on the same day of admission. The mean hospital stay was 5 ± 3.23 hours with no significant difference between both groups. There is significant statistical difference between the studied groups as regards operative time (Table 2). Three cases developed hydrocele in the early postoperative follow-up period in group A, while in group B, postoperative hydrocele was reported in 5 cases. However, all cases responded well to conservative management within 3 weeks (Table 3). Over a mean follow-up period of 24 months (range of 16–30 months), the recurrence rate was 0.8% (one case) in group A, whereas in group B recurrence rate was 2.4% (3 cases) (Table 3).

In group A, there were no cases of iatrogenic ascent of the testis, while in group B 4 cases (4.35%) developed iatrogenic ascent of the testis.

**Figure 3:**
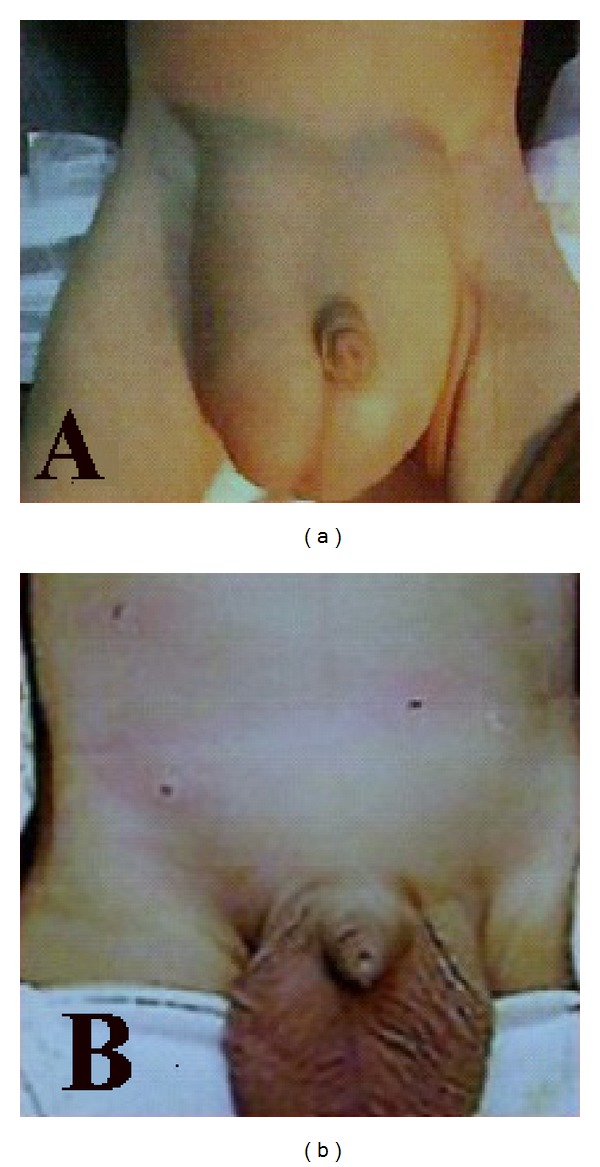
(a) Bilateral huge inguinal hernia. (b) Postoperative view.

**Figure 4:**
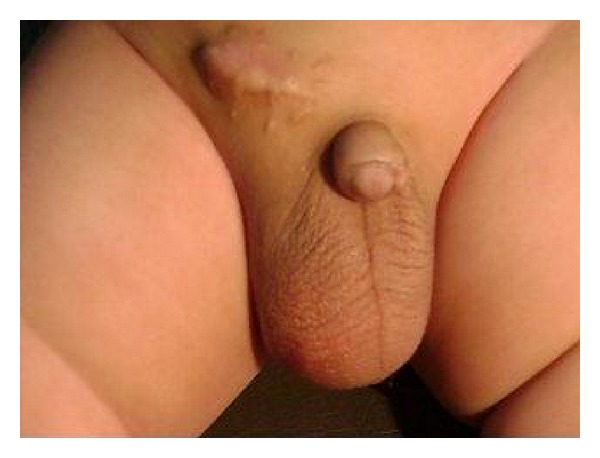
Right inguinal hernia postoperative view with ugly scar.

The early cosmetic results for bilateral cases were excellent (Figures 3(a) and 3(b)). At a follow-up examination more than 6 months later, there were practically no visible scars in group A, while in group B 5 cases had ugly scars as reported by parents (Figure 4). The umbilical scars were not visible in all of the patients of group A.

**Figure 5:**
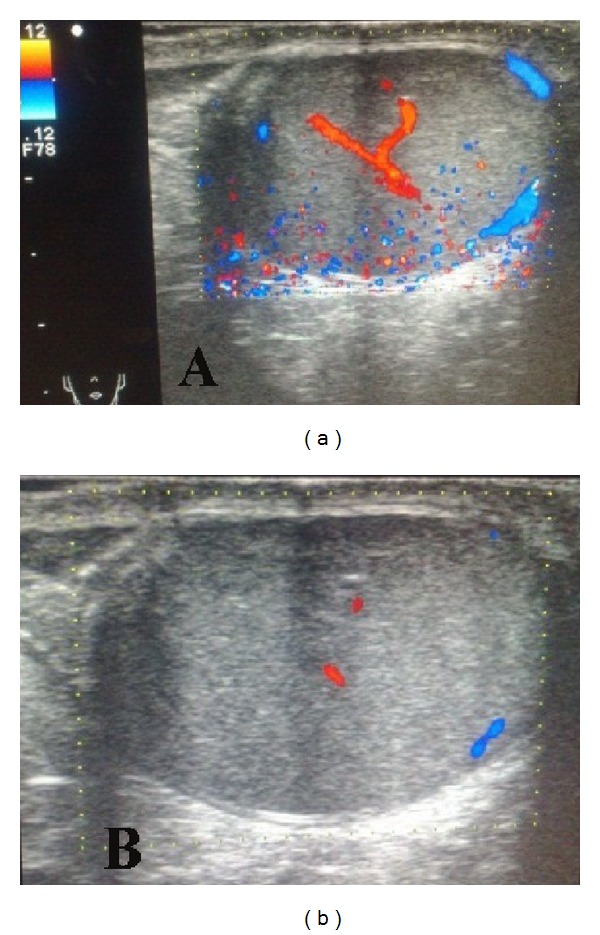
(a) Testicular Doppler U/S showed no signs of ischemia with good blood flow. (b) Testicular Doppler U/S showed poor blood flow.

Concerning the outcome of imaging assessment, in group A, there was no significant difference in values of perfusion and size of the testis between preoperative, early postoperative, and late postoperative periods (Figure 5(a)). While in group B; 3 cases (3.3%) had significant diminution of testicular perfusion and size, indicating atrophy (Figure 5(b)).

Duplex scan was performed for all male cases preoperatively and postoperatively for detection of significant changes of testicular blood flow. RI index was calculated, using paired *t*-test, and *P* values were obtained in group A.

Table 4 clearly shows that there are significant differences (increase of testicular volume) between preoperative and late postoperative volumes of testis units on the operated side in group A, while in group B it clearly shows that there are significant differences (decrease of testicular volume) between preoperative and late postoperative volumes of testis units on the operated side.

The ratio *v* was more than 75% in all cases of group A. RI was less than 0.7 in all cases of group A (no atrophy) as shown in Table 5. The ratio *v* was less than 75% in 3 cases of group B. RI was more than 0.7 in 3 cases of group B (atrophy) as shown in Table 5.

## 5. Discussion

 In children, the standard surgical treatment of IH is limited to division and ligation of the hernial sac at the IIR without narrowing the ring [5]. The internal ring normally is reached by dissecting the hernial sac from the cord structures. Open herniotomy is an excellent method of repair in the pediatric population. However, it has the potential risk of injury of the spermatic vessels or vas deferens, hematoma formation, wound infection, iatrogenic ascent of the testis, testicular atrophy, and recurrence of hernia. It also carries the potential risk of tubal or ovarian damage which may cause infertility [12–14].

Laparoscopic approach is rapidly gaining popularity with more and more studies validating its feasibility, safety, and efficacy [5, 15].

Advantages of laparoscopic inguinal hernia repair include excellent visual exposure, the ability to evaluate the contralateral side, minimal dissection and avoidance of access trauma to the vas deferens and testicular vessels, iatrogenic ascent of the testis, and decreased operative time especially in recurrent and obese cases [3, 5]. However, Alzahem claimed that he is unable to identify any clear benefit of laparoscopic inguinal herniotomy over OH apart from reduction in metachronous hernia development and shorter operative time for bilateral cases [16].

Laparoscopic hernia repair in children is known to take longer operative time than OH. Many reports showed that it ranged from 20 to 74 minutes [5, 17–19]. However, the operative time is reduced with experience. It is well documented that the limiting step in laparoscopic hernia repair is the intracorporeal suturing of the IIR [2, 5]. In OH, time is consumed in gaining access, obtaining adequate exposure, in localizing and isolating the sac from the cord structures. In laparoscopic surgery, approaching the hernial defect from within the abdomen, makes the area of interest bloodless, and the magnification renders anatomy very clear, making surgery precise [13, 15, 20]. With growing experience and use of refinements, such as hydrodissection and needle sign, operative time does come down. Chan and Tam found that laparoscopic surgery is marginally quicker (5 min), but this difference appears insignificant, both statistically and in practice [18].

In our series the operative time is less than that reported in the literature as we use an easy simple and rapid technique for repair of IH using RN which can be done with far great ease in a very short time. Also, we used the extracorporeal suture ligation which is less time consuming [21].

Different laparoscopic techniques for repair of IH in children were reported in the literature. Schier (1998) used 2 mm instruments without a trocar for intra-abdominal suturing of the open inguinal rings in 25 girls by the placement of two Z-sutures with good results [17]. Bharathi et al. stated that SEAL resulted in marked reduction of operative time than TNH technique (unilateral, 15 versus 25 minutes, and bilateral, 25 versus 40 minutes). They added that avoiding the vas deferens and testicular vessels during SEAL repair in males may leave a small gap at the internal ring as well as leaving the hernial sac in situ, which has the potential to contribute to a higher incidence of hydrocele and recurrence in male patients [8, 21]. Yang et al. reported that laparoscopic herniorrhaphy is superior to open herniotomy in the repair of bilateral IH and lower rate of metachronous contralateral hernia, with similar operative time for unilateral hernias, length of hospital stay, recurrence, and complication rates [22]. Endo and Ukiyama introduced the Endoneedle that is designed specifically for laparoscopic extraperitoneal closure of the patent processus vaginalis [23]. Lee and Liang performed microlaparoscopic high ligation in 450 patients with good results. They reported no complications of the surgery and a remarkably low recurrence rate (0.88%) [5].

Marte et al. stated that the incision of the peritoneum lateral to the internal inguinal ring and the W-shaped suture, compared to the sole W-shaped suture, is safe and effective in preventing hernia recurrence [24].

Open herniotomy in children has been reported to have recurrence rates of 0.8–3.8% [8]. While in laparoscopic hernia repair it is ranged from 0.7% to 4.5%. That is may be due to the presence of skip areas during placement of purse-string sutures as well as the tension resulting from intracorporeal knotting particularly in closure of large defects. The critical steps of hernia sac neck transaction at the IIR were not achieved in many laparoscopic procedures unlike during OH. Thus, transient or persistent hydrocele was unavoidable after these laparoscopic techniques. Tsai et al. and others dissected and transected the neck of the sac at IIR to be followed by a suture closure, with this being a faithful reproduction of the inguinal approach [24–26]. They claimed that leaving the hernial sac in continuity without disconnection at IIR may be the cause of subsequent recurrence and hydrocele formation. Ozgediz et al. and Bharathi et al. stated that avoiding the vas deferens and gonadal vessels during subcutaneous endoscopically assisted ligation repair in males may leave a small gap at IIR as well as leaving the hernia sac in situ, which has the potential to contribute to a higher incidence of recurrence in male patients [15, 21]. Technical modifications including injection of saline to lift up the peritoneum, the placement of single suture with complete encirclement of the sac, and disconnection of the hernial sac at IIR have been proposed to reduce the recurrence rates [27].

Yang et al. [22] in their meta-analysis stated that the recurrence rate of laparoscopic hernia repair was higher than OH in 2 studies [9, 28], lower in 3 studies [8, 29, 30], and equal (zero) in 2 studies [3, 10]. In the present study, recurrence rate was 0.8% in group A at one-year followup, while in group B the recurrence rate was 2.4%. The recurrence rate in the group A is lower than that reported in the literature that is because we started laparoscopic hernia repair in our unit after gaining good experiences in different laparoscopic procedures. Complete encirclement of the neck of the sac at the IIR with piercing of the peritoneum twice by RN may add fixation of the suture at this level which prevents migration of the suture distally preventing recurrence. It also, may result in creation of adhesions of the sac minimizing hydrocele formation. Laparoscopic approach was conducted for all recurrent hernias in this study as recommended by others [13, 31].

 The natural history of the PPV in infants remains a controversial topic. Prior studies indicate that 40% of PPVs close spontaneously by two months of age and 60% by 2 years of age; however, the risk of incarceration is highest during infancy [32]. While in some other series PPVs less than 2 mm were not closed [6]. Our approach has been to ligate all PPVs to avoid the development of metachronous hernia. However, more studies are needed to clarify this point.

 For many years, the possible risks of testicular atrophy (0.7–13%), spermatic vessel injury (1.6%), and nerve injury (5–15%) with routine contralateral exploration and repair of PPV in children who have primary unilateral inguinal hernia have been debated [33]. However, in this laparoscopic era, routine exploration and repair of PPV could be a new concept of IH treatment for the following reasons. First, the advantage of laparoscopic hernia repair is the clear and direct view of the vital cord structures that makes dissection of these structures safe and easy. In addition, the incidence of testicular atrophy is so rare in laparoscopic hernia repair because of the multiple collateral circulations of the testis, which makes dissection at IIR level extremely safe even in patients with previous inguinal surgery [34, 35]. Second, the well-known complications with open repair such as iatrogenic cryptorchidism, tethering of the testis and wound infection are almost not seen with laparoscopic repair. Surana and Puri stated that the incidence of iatrogenic ascent of the testis after groin exploration for inguinal herniotomy is 1.2% [36]. A total of 173 boys with previous unilateral inguinal herniotomy were subjected to clinical and U/S examination after a mean postoperative period of 31.68 months. One boy (0.58%) had a more than 50% and 10 boys (5.8%) had a more than 25% decrease in testicular volume on the operated side when compared with the nonoperated side [37].

**Figure 6:**
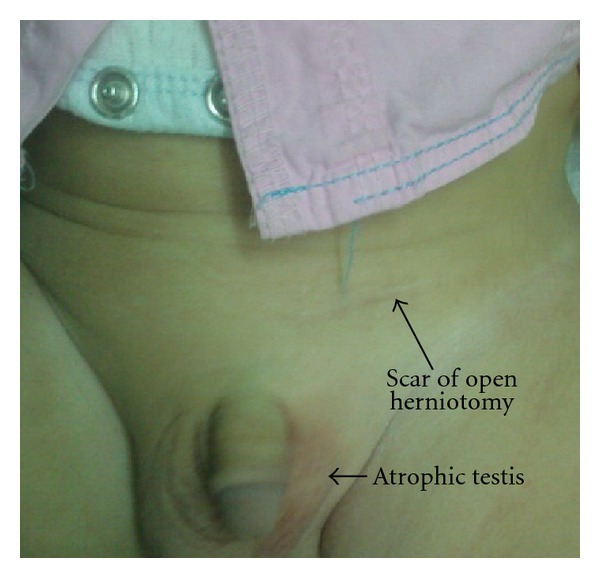
Left testicular atrophy after open herniotomy.

 In our study, no single case of testicular atrophy or iatrogenic ascent of the testis was reported in group A, while in group B 3 cases of testicular atrophy were reported (Figures 5 and 6). Regarding iatrogenic ascent of the testis, no single case was reported in group A, while in group B, 4 cases developed iatrogenic ascent of the testis and the difference is statistically significant. Nagraj et al. reported six cases (2.7%) of testicular atrophy after OH (four of the six patients presented with an incarcerated hernia). There were six cases of iatrogenic ascent of the testis requiring subsequent orchidopexy (2.7%) [38]. Barqawi et al. reported testicular atrophy in 2 cases (1%) after open surgery [34].

Cosmoses, five-millimeter and 3 mm incisions in group A were, indeed, cosmetically more appealing compared with 2 cm incisions in OH group B (Figures 3 and 4). All parents were satisfied with the cosmetic results of group A.

## 6. Conclusion

Our series supports the finding of other series that laparoscopic assisted inguinal hernia repair by RN is feasible safe and rapid technique. It resulted in marked reduction of operative time, low rate of recurrence, no testicular atrophy, no iatrogenic ascent of the testis, and excellent cosmetic results. Complications are minimal though long-term followup will be needed to determine the validity of these results.

## Figures and Tables

**Table 1:** The demographic data for the two groups.

Groups	Group A		Group B		Total	*P* value
	Number	%	Number	%		
Sex						
(i) Male	38	30.4	92	73.6	179 (71.6%)	0.48**
(ii) Female	87	69.6	33	26.4	71 (28.4%)	

Age/month						
(i) 1–12	58	46.4	50	40	95 (38%)	0.80**
(ii) 12–24	45	36	55	44	113 (45.2%)	
(iii) 24<	22	17.6	20	16	42 (16.8%)	

Presentation						
(i) Unilateral in obese child	25	20	28	22.4	53 (21.2%)	0.18**
(ii) Bilateral	44	35.2	48	38.4	52 (20.8%)	
(iii) Recurrent inguinal hernia	12	9.6	15	12	27 (10.8%)	
(iv) Inguinal hernia with umbilical hernia	18	14.4	22	17.6	40 (16%)	
(v) Inguinal hernia with questionable other side	26	20.8	12	9.6	38 (15.2%)	

**Insignificant.

**Table 2:** Distribution of the studied groups according to operative time.

Groups	Group A (mean SD)	Group B (mean SD)	*P* value
Unilateral and recurrent unilateral	7.6 ± 3.5 minutes	12.8 ± 4.5 minutes	<0.001*
Hernia in obese child	9.2 ± 4.6 minutes	14.3 ± 3.6 minutes	<0.001*
Bilateral	11.4 ± 2.7 minutes	21.9 ± 7.2 minutes	<0.001*

*Significant.

**Table 3:** Postoperative complications in the studied groups.

Groups	Group A		Group B		*P* value
	Number	%	Number	%	
Hydrocele	3/87	2.4%	5/92	5.4%	0.52**
Recurrence	1/125	0.8%	3/125	2.4%	0.31**
Iatrogenic ascent of the testis	0/87	0%	4/92	4.35%	0.049*
Testicular atrophy	0/87	0%	3/92	3.3%	0.089**
Ugly scar	0/125	0%	5/125	4.0%	0.024*

*Significant, **insignificant.

**Table 4:** Evaluation of volume of testis in males of both groups.

Groups	Group A (*n* = 87)			Group B (*n* = 92)		
	Mean volume	Volume range	SD	Mean volume	Volume range	SD
Preoperative	1.34	1.01–1.41	0.03*	1.35	0.89–1.49	0.05*
Late postoperative	1.36	1.21–1.86	0.05*	1.31	0.22–1.56	0.12*

Statistics	Paired (*t*) = 4.73, *P* value < 0.001*			Paired (*t*) = 6.36, *P* value < 0.001*		

*Significant.

**Table 5:** Duplex evaluation of centripetal artery in males of both groups.

Groups	Group A (*n* = 87)			Group B (*n* = 92)		
	Mean (RI)	RI range	SD	Mean (RI)	RI range	SD
Preoperative	0.48	0.41–0.52	0.03*	0.48	0.41–0.52	0.029
Early postoperative	0.47	0.40–0.52	0.031*	0.47	0.40–0.52	0.031
Late postoperative	0.46	0.40–0.49	0.032*	0.50	0.40–0.78	0.037

Statistics	Paired (*t*) = 75.0, *P* value < 0.001*			Paired (*t*) = 3.02, *P* value = 0.003*		

* Significant.
